# Silicon Nanowires: A Breakthrough for Thermoelectric Applications

**DOI:** 10.3390/ma14185305

**Published:** 2021-09-14

**Authors:** Giovanni Pennelli, Elisabetta Dimaggio, Antonella Masci

**Affiliations:** Dipartimento di Ingegneria dell’Informazione, Università di Pisa, Via G.Caruso, I-56122 Pisa, Italy; elisabetta.dimaggio@ing.unipi.it (E.D.); a.masci1@studenti.unipi.it (A.M.)

**Keywords:** silicon nanowire, thermoelectricity, thermal conductivity, figure of merit

## Abstract

The potentialities of silicon as a starting material for electronic devices are well known and largely exploited, driving the worldwide spreading of integrated circuits. When nanostructured, silicon is also an excellent material for thermoelectric applications, and hence it could give a significant contribution in the fundamental fields of energy micro-harvesting (scavenging) and macro-harvesting. On the basis of recently published experimental works, we show that the power factor of silicon is very high in a large temperature range (from room temperature up to 900 K). Combining the high power factor with the reduced thermal conductivity of monocrystalline silicon nanowires and nanostructures, we show that the foreseen figure of merit ZT could be very high, reaching values well above 1 at temperatures around 900 K. We report the best parameters to optimize the thermoelectric properties of silicon nanostructures, in terms of doping concentration and nanowire diameter. At the end, we report some technological processes and solutions for the fabrication of macroscopic thermoelectric devices, based on large numbers of silicon nanowire/nanostructures, showing some fabricated demonstrators.

## 1. Introduction

Thermoeletric devices can convert heat directly into electrical power, and vice versa. They are compact and reliable, because they do not have any moving mechanical parts, and potentially they have a broad range of applications: energy recovery and green energy harvesting; energy micro-harvesting (scavenging) for the capillary supply of small systems, such as sensor nodes for Internet of Things (IoT); powering of systems in remote and harsh environments typical, for example, of spatial exploration; localized and optimized cooling of small systems, where the reliability and the compactness can play a fundamental role. Unfortunately, at the present state of the art, all these potentialities offered by thermoelectric devices [1] (Thermoelectric Generators, TEG, and Thermoelectric Coolers, TEC) are limited by the available materials with thermoelectric properties good enough for an acceptable thermal to electrical conversion efficiency. The parameter expressing the thermoelectric properties of a material is the dimensionless figure of merit ZT, where *T* is the absolute temperature and $\frac{Z=S^{2}\sigma }{k_{t}}$, *S* is the Seebeck coefficient, σ is the electrical conductivity and $k_{t}$ is the thermal conductivity. A good thermoelectric material should have ZT as high as possible, that means *S* and σ as great as possible, and $k_{t}$ as low as possible. However, more factors must be taken into account in the evaluation of materials for thermoelectric conversion: (1) Sustainability: devices for green application must be manufacturable with materials that are not toxic or polluting. (2) Availability and costs: wide scale applications need to be based on abundant and cheap materials. (3) Technical feasibility: a material with good transport properties is only a starting point; making a device requires the possibility to tailor the doping concentration, either *n* and *p*, to make good electrical contacts and so on. (4) Stability on long times and, in particular, in a large temperature range: for the second principle of thermodynamics, machines for conversion of heat into other energy forms must exploit high temperature differences to achieve high conversion efficiencies. Hence, materials able to maintain good thermoelectric properties on large temperature ranges are required. At the current state of the art, most of the commercial TEG/TEC are based on a tellurium compound, which is a poisoning and a very rare element: its abundancy is less than 0.001 ppm on the Earth’s crust, comparable with that of platinum. Standard TEG/TEC devices, based on bismuth telluride, have good thermoelectric characteristics, with ZT approaching 1, but in a limited temperature range around room temperature. Its thermoelectric properties drop very fast for temperatures over 150 °C. Research is focusing on a large plethora of materials with good thermoelectric properties [2,3]. In this article, we focus on the thermoelectric properties of silicon [4,5]. Silicon satisfies all the points requested for an excellent material for the fabrication of devices, being sustainable, biocompatible, abundant (the Earth’s crust is essentially made of silicon). It can be said that silicon is the most investigated material ever, because of its large use in the microelectronic industry. A huge amount of data is available about doping processes and electrical conductivity; there are many studies on interfaces between silicon and a large range of materials, for the fabrication of contacts, and so on. The key point is the nanostructuration of silicon: silicon nanowires showed a thermal conductivity as small as 2 W/(m K). This overcomes the Achilles heel of silicon for thermoelectric applications, that is its high thermal conductivity: in its bulk form, $k_{t}$ for silicon is 148 W/(m K). Combined with the high power factor value, this strong reduction of the thermal conductivity places silicon in an excellent position as a material with a very high value of ZT. At first, in Section 2, we report the figure of merit of silicon as a function of temperature, achieved by crossing available experimental measurements, and we show that ZT is very high on a large temperature range if the correct parameters (doping concentration and nanowire size) are chosen. The price to be payed for the exploitation of these excellent thermoelectric potentialities of silicon is the development of techniques for its nanostructuration, which need to be reliable and low-cost. For this reason, as a second point of our work, in Section 3, we discuss and report some of these techniques, based on standard silicon technologies, and we show some demonstrators that we developed.

## 2. Thermoelectric Properties of Nanostructured Silicon

### 2.1. Electrical Conductivity, Seebeck Coefficient and Power Factor

On the basis of available experimental measurements, we evaluate the power factor of silicon as a function of the doping concentration, both *n* and *p*, and of the temperature. To this end, we need to extrapolate the electrical conductivity and the Seebeck coefficient.

The electrical conductivity of silicon has been largely investigated in the past, due to the relevance of silicon for the fabrication of integrated circuits. Data on the electrical conductivity as a function of doping concentration and temperature, and for several doping species (Boron, Phosphorous, Arsenic), are largely available. Several semi-empirical models, which give a good fit of conductivity measurements, have been developed. To be mentioned are the Arora’s formulas [6], available both for *n* and for *p* doping, and the Masetti’s model [7] in its extended version developed by the group of the University of Bologna [8]. These semi-empirical models of silicon conductivity are very reliable, and they are largely used by several computer aided design (CAD) codes for electron device simulation, such as Synopsis. The electrical conductivity of silicon nanowires can be affected by the surface scattering, in particular for nanowires smaller than 20 nm (see ahead).

The Seebeck coefficient of silicon is not as much investigated as the electrical conductivity. One of the first studies was performed in 1955 by Geballe et al. [9], which reported the Seebeck coefficient for several *n* and *p* doping concentrations, for temperature up to 300 K. The Seebeck coefficient of *n*-doped silicon has been reported by Brinson [10] in 1970 for temperatures in the range 0–300 K. More recently, in 2013, Stranz et al. [11] reported the Seebeck coefficient for *n* a *p* heavily doped silicon, for temperatures up to 700 K. Ohishi et al. [12] (2015) reported the electrical conductivity and the Seebeck coefficient for heavily boron and phosphorous doped silicon, for temperatures up to 1000 °C. In 2017, Bennett [13] made a study of the Seebeck coefficient at room temperature, for different *n* doping species (phosphorous, arsenic and antimony), as a function of the Hall carrier concentration. Given a doping concentration, a rough approximation of the Seebeck coefficient can be achieved with the Stratton formula:

(1) $$S=-\frac{k_{B}}{e}\left(\frac{5}{2}-\log\frac{n}{N_{C}}\right)$$

where $k_{B}$ is the Boltzmann constant, *e* is the elemental charge, *n* is the electron density in the conduction band, which, at room temperature, is approximately the doping concentration, and $N_{C}=N_{C}\left(T\right)$ is the equivalent density of states in the conduction band for silicon, $N_{C}=2.8\times 10^{24}$ m${}^{-3}$ at room temperature. For a better extrapolation of the Seebeck coefficient as a function of the doping concentration, we propose to use a logarithmic fit [14] of the available experimental data.

**Figure 1:**
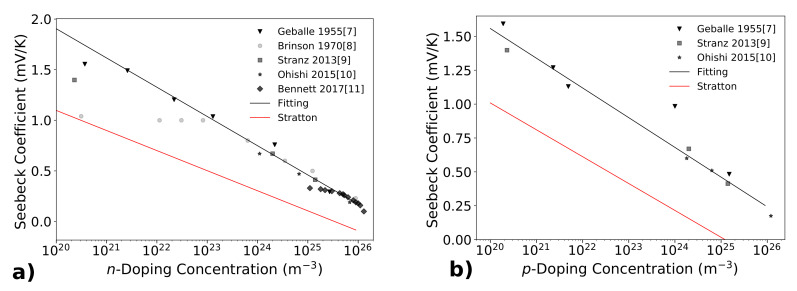
Available experimental data of the Seebeck coefficient are reported both for *n*-doping (Panel (**a**)) and for *p*-doping (Panel (**b**)). The logarithmic fit (see text) is also shown. The red lines show the Seebeck coefficient calculated with the Stratton formula (both for *n* and for *p* doping): experimental measurement give higher values of the Seebeck coefficient. Panel (**a**) is a modification of the figure published on [14].

Figure 1a shows the experimental values of the Seebeck coefficient at room temperature for *n* doped silicon (in absolute value, it is negative for *n* doping). Data on *n* doping are reported by Geballe [9], Brinson [10], Stranz [11], Ohishi [12] and Bennett [13]. The best log fit shown in the graph resulted:

(2) S(n)=−0.000125×log(n)+0.00768V/K

where *n* is the electron concentration in m${}^{-3}$ (International System of Units). The Seebeck coefficient evaluated with the Stratton formula was also reported, which in general gives an underestimation with respect to the experimental data. For *p* doping, there are less experimental data, reported by Geballe [9], Stranz [15] and Ohishi [12]. These data are reported in Figure 1b, together with the log fit, which resulted as:

(3) $$S\left(p\right)=-9.523\times 10^{-5}\times \log\left(p\right)+0.0059434$$

The Seebeck coefficient evaluated by means of the Stratton formula is also reported.

The estimation of the Seebeck coefficient through Formulas (2) and (3) can be used for the evaluation of the power factor $S^{2}\sigma$ as a function of doping. In this work, we used the Arora’s formula for the evaluation of σ.

**Figure 2:**
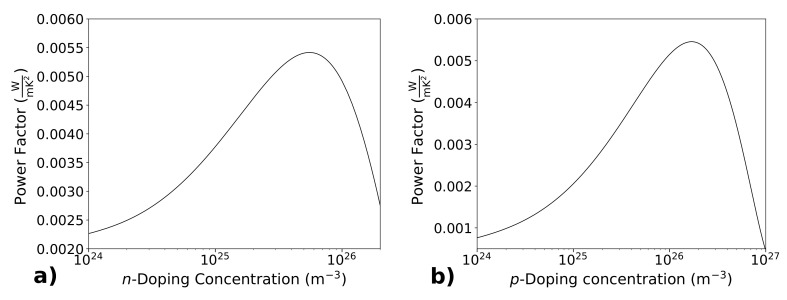
The room temperature power factor $S^{2}\sigma$ is reported as a function of the doping concentration, both for *n* (Panel (**a**)) and for *p* type silicon (Panel (**b**)). An optimal doping for the maximum power factor is established both for *n* ($n=5.5\times 10^{25}$ m${}^{-3}$) and for *p* ($p=1.2\times 10^{26}$ m${}^{-3}$) type silicon. Panel (**a**) has been reprinted from [14] with permission.

Figure 2 shows the silicon power factor at 300 K, as a function of the doping concentration, both for *n*-doped (Panel (a)) and *p*-doped (Panel (b)) silicon (Panel (a) has been taken from [14] with permission). It is well known that the Seebeck coefficient and the electrical conductivity have an opposite trend with the increasing of doping concentration: the Seebeck coefficient decreases with the doping, meanwhile the electrical conductivity increases. Therefore, the power factor at first increases with the doping, when the weight of the increasing of σ overcomes that of *S*, and then decreases for very high doping, where the decreasing of *S*, which appears squared in the power factor, begins to predominate. As a consequence, the doping concentration must be tailored to get the maximum power factor. For *n*-doped silicon, at room temperature, the maximum power factor resulted 0.0054 W/mK${}^{2}$ for a doping concentration $n=5.5\times 10^{25}$ m${}^{-3}$ ($5.5\times 10^{19}$ cm${}^{-3}$). For *p*-doped silicon, the maximum power factor resulted 0.0054 W/mK${}^{2}$ for a doping concentration $p=1.7\times 10^{26}$ m${}^{-3}$ ($1.7\times 10^{20}$ cm${}^{-3}$). These results for the power factor are quite high, comparable and even better of those achieved with the best thermoelectric materials.

**Figure 3:**
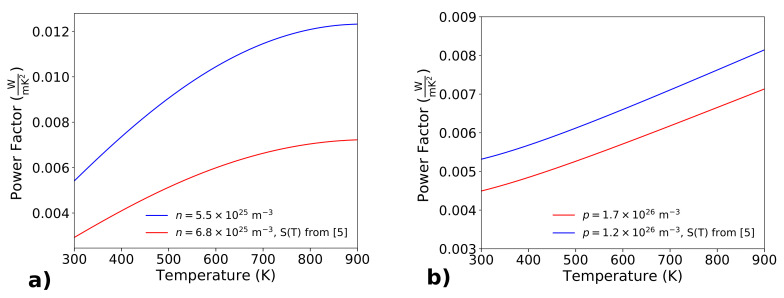
The power factor of *n* (Panel (**a**)) and *p* (Panel (**b**)) doped silicon is reported as a function of temperature. The blue curves concern the doping concentration, which maximizes the power factor at room temperature, the red curves have been calculated considering S=S(T) experimentally determined by Ohishi [12] for doping concentrations different from that for the maximum power factor.

An important evaluation that must be done is the power factor as a function of the temperature, especially for temperatures higher than 300 K, where thermoelectric devices can find useful applications. Most of the semi-empirical formulas for the evaluation of the electrical conductivity, such as the Arora one, gives its dependence on the temperature. Instead, very few data are available for the temperature dependence of the Seebeck coefficient S=S(T). Geballe [9] reports *S* in the temperature range 300–1000 K, but for low values of doping concentration (around 10${}^{20}$ m${}^{-3}$). Both Stranz [15] and Ohishi [12] report *S* for doping concentration between 10${}^{20}$ m${}^{-3}$ and 10${}^{26}$ m${}^{-3}$. In particular, Ohishi [12] reports *S* for $n=6.8\times 10^{25}$ and $p=1.7\times 10^{26}$ m${}^{-3}$, for temperatures up to 1000 K. These values are very interesting, because they are very close to those for which both *n* and *p* type silicon show the maximum power factor at room temperature. It must be noted that, regarding *n* type silicon, the increasing of temperature brings an increase of the electrons at higher energies, and hence the absolute value of the Seebeck coefficient increases. This is true up to temperatures for which the concentration of the thermally generated electron-hole pairs becomes comparable with that of the electrons coming from the donor level. A further increasing of the temperature produces a decreasing of the Seebeck coefficient, because the electrical conduction is dominated by a comparable number of electrons and holes, which give an opposite contribution to the Seebeck coefficient. The same behavior can be noticed for *p* type silicon. This is visible in the graphs of Figure 8 of Geballe [9], where it is shown that *S* decreases for temperature greater than 500 K, for doping concentration of about 10${}^{20}$ m${}^{-3}$. In Figure 2, Ohishi [12] reports S=S(T) for a few doping concentrations: for doping concentrations of about 10${}^{24}$ m${}^{-3}$, *S* at first increases, both for *n* and for *p* doping, and then *S* begins to decrease for temperatures higher than 800 K. However, the maximization of the power factor is achieved with very high doping concentrations, above 10${}^{25}$ m${}^{-3}$. For these concentration values, the Seebeck coefficient increases from room temperature to temperatures in excess to 1000 K. As a reference number for the thermally generated electron-hole pairs, we can consider the intrinsic carrier concentration $n_{i}$, which is about 10${}^{16}$ m${}^{-3}$ at room temperature, and increases to 10${}^{24}$ m${}^{-3}$ for T=800 K. The temperature must exceed 1400 K to have $n_{i}=10^{26}$ m${}^{-3}$. Figure 3 shows the power factor $S{\left(T\right)}^{2}\sigma \left(T\right)$ as a function of temperature for *n* (Panel (a)) and *p* (Panel (b)) doped silicon, for different values of doping concentrations. In Panel (a), the red curve shows the power factor $S{\left(T\right)}^{2}\sigma \left(T\right)$ for $n=6.8\times 10^{25}$ m${}^{-3}$. For this concentration, experimental values for S=S(T) have been made available by Ohishi [12] (see Figure 2). For Figure 3a a parabolic fitting of the Ohishi’s data has been used:

(4) $$S\left(T\right)=S\left(T=300\;K\right)\left[1+a\left(T-300\right)+b{\left(T-300\right)}^{2}\right]$$

where S(T=300K)=0.181 mV/K, and $a=2.78\times 10^{-3}$, $b=-1.81\times 10^{-6}$. The blue curve shows the power factor for a doping concentration of $n=5.5\times 10^{25}$ m${}^{-3}$, for which it is maximum at room temperature. In the Formula (4) S(T=300K)=0.259 mV/K, evaluated by the Formula (2), has been used; this value is very close to the experimental data of Bennett [13], which measured S=0.26 mV/K for $n=5.3\times 10^{25}$ m${}^{-3}$. For *a* and *b* the same values achieved with the doping concentration $n=6.8\times 10^{25}$ m${}^{-3}$ have been taken, assuming that they do not change very much for doping concentrations around that number. More experimental measurements should be performed to confirm this assumption. It must be noted that the value of S(T=300K)=0.181 mV/K achieved by Ohishi [12] is not consistent with the fit for *S* at room temperature (Formula (2)), which gives S=0.244 mV/K for $n=6.8\times 10^{25}$ m${}^{-3}$. >From the curve, we can see that the power factor increases with temperature: from one side the electrical conductivity decreases with temperature for the increasing of the scattering rate; on the other side, *S* increases with temperature, and it predominates as it appears squared in the power factor. The power factor is $5.4\times 10^{-3}$ W/mK${}^{2}$ at room temperature, and reaches an astonishing value of $12\times 10^{-3}$ W/mK${}^{2}$ at 900 K. A similar behavior is achieved for *p* doped silicon (Figure 3b), where together with the values based on the measured S=S(T) for $p=1.7\times 10^{26}$ m${}^{-3}$ (red curve), is reported that for $p=1.2\times 10^{26}$ m${}^{-3}$, which maximizes the power factor at room temperature. Furthermore, in the case of *p* doped silicon, the power factor increases in the temperature range 300–900 K.

It must be noted that, even if the power factor of silicon is already quite high, it can be further increased implementing techniques of energy filtering [16,17]. As an example, thanks to energy filtering effects, heavily *p*-doped bulk nanocrystalline silicon [18,19] showed an increase both of the electrical conductivity and of the Seebeck coefficient.

### 2.2. Thermal Conductivity and Figure of Merit

The strong reduction of the thermal conductivity in nanostructured materials [20] has been largely demonstrated. In particular, a conductivity smaller than 10 W/(m K) has been reported in 2003 by Li et al. [21] for monocrystalline silicon nanowires smaller than 30 nm. Since that year, many experimental works have been devoted to the measurement of the thermal conductivity together with other thermoelectric parameters of silicon nanowires and nanostructures [14,22,23,24,25,26,27,28,29,30,31,32,33,34,35]. In 2008, two famous works have been published on Nature Letters: both Hockbaum et al. [23] (who used Metal Assisted Chemical Etching for the production of nanowires) and Boukay et al. [36] reported the thermal conductivity of SiNWs; they positioned one, or very few, nanowires on suspended platforms, completed with microfabricated heaters and thermometers, and both of them achieved a thermal conductivity below 2 W/(m K). These results have been confirmed by macroscopic measurements on a huge number of nanowires, organized in vertical forests [29,34,37]: a thermal conductivity of 4.6 W/(m K) [37] has been measured on nanowire forests with low doping values and with an average nanowire diameter around 80 nm; the thermal conductivity resulted as small as 1.7 W/(m K) for heavily doped $p^{+}$ nanowire forests [34], whose average nanowire diameter is of 40 nm. Several theoretical works [38,39] demonstrated that the enhancement of the phonon scattering, due to the roughness of the nanowire walls, limits the phonon propagation, reducing the thermal conductivity. The phonon mean free path, at room temperature, is of the order of several tens of nanometers, and hence nanowires narrower than 100 nm represent an almost 1D conductor for phonon transport, which can freely move only in one direction. Several experimental works confirmed this strong relation between thermal conductivity reduction, nanowire diameter and surface roughness of nanowires [25,26,27]. In particular, the thermal conductivity of top-down fabricated smooth nanowires, 100 nm wide, resulted as 40 W/(m K) [24]. Lim et al. [27] demonstrated that, in Si nanowires with diameter of about 70 nm, the thermal conductivity drops from more than 20 W/(m K) to less than 2 W/(m K), increasing the surface roughness [27]. For this reason, values of thermal conductivity smaller than 5 W/(m K) have been measured with Si nanowires fabricated by Metal Assisted Chemical Etching [23,30,34,36,37], which produces nanowires with very rough surfaces. Meanwhile, smooth nanowires fabricated by top-down lithography and etching [24] or by CVD [35,40] always showed thermal conductivities of a few tens of W/(m K).

The thermal conductivity of thin monocrystalline silicon membranes, which are silicon structures where one dimension is very small if compared with the other two (almost 2D structures), has also been measured. In this case, we can think of a 2D phonon transport, and the thermal conductivity in the film plane is reduced for the scattering on the top and bottom surfaces of the film. A strong reduction of thermal conductivity has been measured on Si membranes thinner than 200 nm [41,42]. In particular, in our previous paper [42], we demonstrated a strong relationship between thermal conductivity and surface roughness of Si nanomembranes. The surface roughness has been characterized by AFM measurements, and a dedicated 3ω technique, applied to suspended nanomembranes, has been used for in-plane thermal conductivity measurements [43]. A thermal conductivity of 17 W/(m K) has been measured on a 120 nm thick Si nanombembranes.

**Figure 4:**
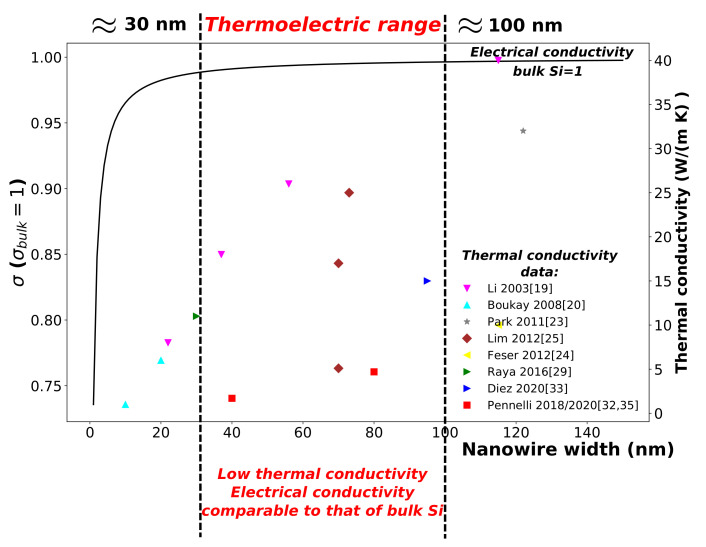
The electrical conductivity for $n=5.5\times 10^{25}$ m${}^{-3}$, normalized with respect to the bulk value, is reported as a function of the nanowire width (continuous curve). Furthermore, the experimental measurements of the thermal conductivity (scatters) are reported. For diameters between 30 nm and 100 nm (roughly), the electrical conductivity is comparable with that in the bulk, the thermal conductivity results were instead strongly reduced for sufficiently rough nanowires.

As anticipated by Dresselhaus and co. [44,45], the benefits of nanostructuring consists both in the decreasing of the thermal conductivity and in the increasing of the Seebeck coefficient *S*. However, nanowires narrower than 5 nm are required for an effective increasing of the Seebeck coefficient [46], and at these dimensions the electrical conductivity is strongly reduced for the scattering of the charged carriers on the nanowire walls [5]. For a good electrical conductivity, comparable to that of bulk silicon, nanowires wider than 30 nm should be considered. On the other hand, a width smaller than 100 nm is sufficient for a strong reduction of the thermal conductivity. In Figure 4, it is shown that the optimal diameter of nanowires/nanostructures for good thermoelectric properties is between 30 nm and 100 nm: for this diameter range, the thermal conductivity can be very small if rough nanowires are fabricated, meanwhile the electrical conductivity and the Seebeck coefficient are very close to those of the bulk silicon.

**Figure 5:**
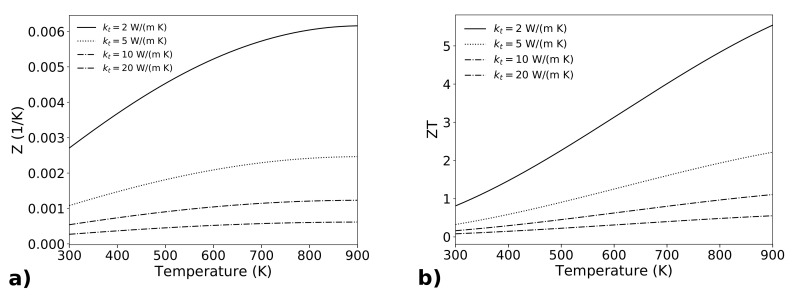
The factor *Z* (Panel (**a**)) and the figure of merit ZT (Panel (**b**)) are reported as a function of temperature, for the optimum thermoelectric doping of *n* type silicon. Different curves consider different values of the thermal conductivity $k_{t}$. The highest values of *Z* are related to $k_{t}=2$ W/(m K), achievable in rough silicon nanowires.

Figure 5 reports the parameter $Z=S^{2}\sigma /k_{t}$ and the figure of merit ZT as a function of temperature *T*. For the power factor $S^{2}\sigma$ the data of Figure 3, for a doping concentration $n=5.5\times 10^{25}$ m${}^{-3}$, have been used. Different values of the thermal conductivity $k_{t}$, which has been considered constant with temperature, have been considered. It has been demonstrated that the thermal conductivity of silicon nanowires decreases with temperature, for temperatures above 300 K [47] up to 700 K. To our knowledge, there are no available data of $k_{t}=k_{t}\left(T\right)$ for temperatures up to 900 K, but it can be assumed that the thermal conductivity further decreases with the increasing of temperature, therefore the data shown in Figure 5 are prudential because they consider $k_{t}$ constant with temperature. In general, it must be noted that the figure of merit ZT increases with temperature even in the case that Z=Z(T) does not change with temperature. Hence, the parameter *Z* (K${}^{-1}$) is a better indicator of the properties of the material, because an increasing of *Z* with temperature really demonstrates the goodness of a material for thermoelectric applications. Figure 5a shows the parameter $Z=\frac{S^{2}\sigma }{k_{t}}$ for silicon, for different values of the thermal conductivity $k_{t}$. The lower value considered is 2 W/(m K), which is reasonably achievable with rough nanowires. Even intermediate values of 5 or 10 W/(m K) give a high *Z* value, always increasing with temperature. For comparison with other materials, the parameter ZT is shown on Panel (b). For a thermal conductivity of 2 W/(m K), and for an optimized doping value, we have ZT=0.8 at room temperature, and an astonishing value of about 5 is achieved at temperatures around 900 K.

## 3. Techniques for All-Silicon Thermoelectric Devices

The main issue for the use of silicon in thermoelectric applications is the development of strategies and techniques for the fabrication of a large number of silicon nanowires and nanostructures, to be interconnected in a suitable way for making possible the thermal and electrical transport. Starting from the enormous background of silicon, developed within the last decades to support the world widespread market of electronic circuits, a large variety of nanostructured silicon thermoelectric devices are being investigated and developed, with the possibility to cover both the aspect of energy harvesting on large scale (macroharvesting) and on millimetric/micrometric scale (microharvesting, or scavenging). The main fabrication strategies currently under investigation are [48,49]: (1) the planar strategy, consisting in the fabrication of many Si nanostructures parallel to the silicon substrate. (2) The vertical strategy, which relies on highly selective etching processes for the definition of Si nanowires perpendicular to a silicon substrate.

### 3.1. On-Chip Si Nanowires/Nanostructures for Energy Scavenging

One of the strengths of silicon thermoelectric nanodevices is that processes for their fabrication can be based on well-assessed integrated circuit fabrication techniques. Micromachining techniques can be used to fabricate nanowires interconnected and placed across microfabricated heat exchangers, to be connected to the hot and cold heat sources. These technologies are suitable for device areas of the order of several millimeters square at maximum, so that they are suitable for microharvesting/energy scavenging. The range of applications is in any case very wide, because on-chip integrated devices can directly produce electrical power from any hot surface, and they could be fabricated side by side with other integrated electronic circuits, providing their supply.

As the typical processes for IC fabrication are planar (electronic devices are fabricated on the surface of a silicon wafer), the best strategy for on-chip thermoelectric devices for energy scavenging is the planar one, also if solutions based on lithography/etching of very short nanowires perpendicular to the silicon substrate have been proposed [50]. The main point of the planar strategy is that it requires the suspension of nanostructures which must interconnect the hot side with the cold side, avoiding any heat conduction through the substrate. Hence, one of the difficult tasks involved with the use of this technique is the connection of these very fragile suspended silicon platforms [51] with the heat sources. On-chip thermoelectric devices can be fabricated by CVD growth (bottom-up approach) of Si, or SiGe, nanowires between a suspended silicon platform, which can be used as hot part of the device, and the body of the chip [52,53,54,55,56] (heat sink, cold part of the device). Millions of nanowires can be simultaneously grown by VLS-CVD [57] through catalyzing gold seeds, deposited by galvanic displacement [58,59]. Therefore, this technique does not require high resolution lithography for the definition of nanowires: it requires only almost standard micromachining techniques, with relaxed lithography, for the fabrication of the platforms and of the electrical contacts. The metal-nanowire electrical contact has been investigated, and very low values of contact resistances have been achieved, by Gaeda and co. [60]. As alternative, suspended silicon platforms can be used to host pn junction made of polysilicon [61,62] or porous silicon [63].

**Figure 6:**
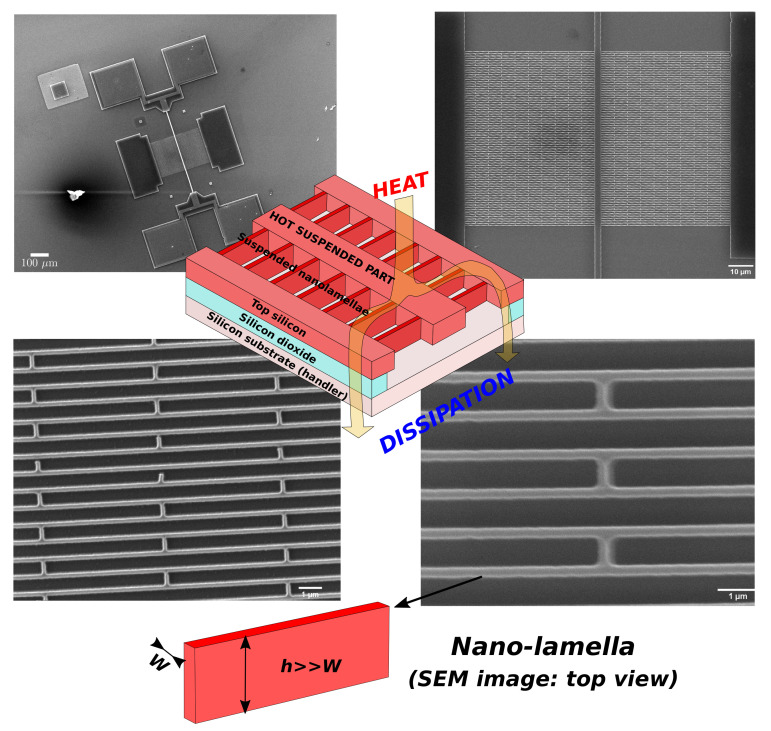
Original SEM micrographs of fabricated prototypes, together with a sketch of these planar thermoelectric devices. These prototypes confirm the feasibility of an all-silicon thermoelectric device, based on large arrays of silicon nanostructures.

A possible alternative approach is to define the nanostructures/nanowires by lithography and etching [64,65] (top down approach), using a suitable design to connect silicon platforms, which can be fabricated simultaneously with the nanostructures. The main advantage of this strategy is the high flexibility in the design of the device: nanostructures can be fabricated and positioned in precise positions and configurations. Another great advantage is that there is crystalline continuity between silicon platforms and silicon nanowires, so that any problem of electrical and thermal contact resistance is avoided: metal contacts can be fabricated in large areas, even not very close to the nanostructures, as silicon is heavily doped (low resistance) for thermoelectric purposes. The challenging point, and main draw-back, of this top-down strategy is that it requires high resolution lithography because the reduced dimensions of the nanostructures rely on the potentialities of the lithographic step. Nanowires narrower than 60 nm arranged in large area networks of the order of mm${}^{2}$ have been fabricated, and the design has been studied so that the large number of interconnections gives a high reliability with respect to nanowire failure [64]. An alternative approach to the use of nanowires is to use silicon lamellae (bidimensional, narrow and tall silicon nanostructures) [66]. They can be fabricated with the large side perpendicular to the silicon substrate, so that a larger number of them can be packed side by side on the same device area. From one side, the reduction of thermal conductivity is less effective (they are bidimentional structures), but on the other side the mechanical stability is increased as also the overall cross-section: this increases both the electrical current (more power) and the heat flux, whit respect to the case of using nanowires with the same width. Figure 6 shows SEM images of a demonstrator based on silicon nano-lamellae fabricated on a Silicon-On-Insulator (SOI) wafer with a top silicon layer 260 nm thick, a buried oxide layer 2 μm thick and a handler layer 500 μm thick. We developed this demonstrator just to prove the feasibility of a large collection of interconnected silicon nanostructures. The lamellae are as narrow as 100 nm and as tall as the thickness of the top silicon layer (260 nm, used for the demonstrator). The large side is perpendicular to the surface, therefore this design allows the packing of a large number of nanostructures, limited only by the width and the pitch between the nano-lamellae. Thicker SOI wafer can be considered: the limit of the width/height ratio is determined by the aspect ratio of the selective plasma etching process, which could reach 100:1 [67,68,69,70,71] (10 μm for a width of 100 nm) if deep reactive etching is used.

### 3.2. Si Nanowires/Nanostructures for Energy Macroharvesting

**Figure 7:**
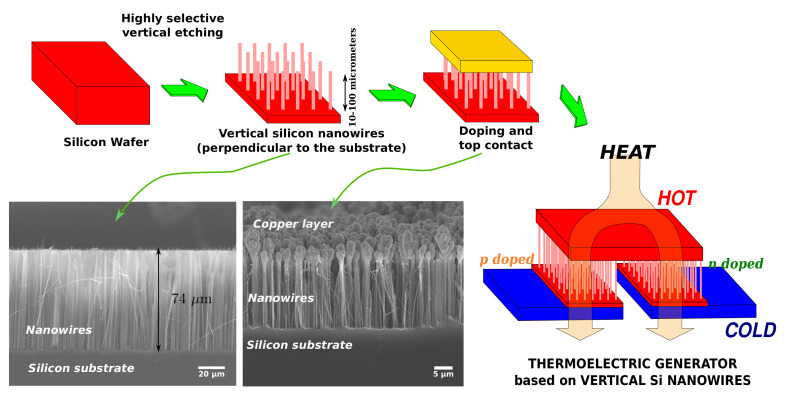
Sketches and SEM micrographs of thermoelectric devices based on silicon nanowires fabricated perpendicular to the substrate.

The use of nanostructured silicon for macroharvesting requires the development of low-cost nanostructuration techniques that can be applied on large areas. This excludes the use of advanced high-resolution lithography, whose costs increase rapidly with the increasing of the surface. This also makes the implementation of planar strategies difficult, because high power output means having high current and hence a very large number of parallel nanostructures, which cannot be fabricated with planar standard CMOS technologies. The reasonable configuration for large-area nanostructured silicon thermoelectric devices consists of nanowires/nanostructures placed perpendicularly to the silicon substrate, which can be fabricated by means of maskless (no lithography), highly selective etching processes, such as metal assisted chemical etching (MACE) [72,73]. Very shortly, it consists in depositing metal nanoparticles, which can be gold, silver [74,75,76] and also ruthenium [77] (investigated very recently), on the top of a standard (and hence low-cost) silicon wafer. Soaking the wafer in an acqueous solution containing an oxidizing agent and HF, the silicon is locally oxidized very close to the nanoparticles (catalyzing agent) and then removed by HF. Therefore, the nanoparticles soak into the silicon wafer leaving monocrystalline silicon pillars all around. The deposition of nanoparticles does not necessarily require expensive lithographic processes, it can be done by galvanic displacement. Nanowires have a random diameter, but the achievable average is of few tens of nanometers, well within the thermoelectric range. The best and more reliable procedure is just to soak a bare silicon wafer in a solution containing hydrofluoric acid (HF) and silver nitrate [72,75,78,79]. In the same step, silver nanoparticles are deposited on the surface and silicon is locally etched by their catalysis. Billions of nanowires per cm${}^{2}$ can be fabricated with this technique: the average diameter is around 80 nm, the length depends on the etching time and can easily reach 100 μm [14], resulting in an impressive aspect ratio (>1000:1). Several works [22,27] used single silicon nanowire, produced by MACE, for the measurement of the thermal conductivity: once fabricated by MACE, a single or very few nanowires have been positioned on suspended platforms for the characterization. However, as shown in Figure 7 a thermoelectric device can exploit the overall forest, hence it is important to measure the thermal conductivity of large area samples. It has been demonstrated that, even if the nanowire diameters are not uniform, the thermal conductivity of the overall nanowire forest is strongly reduced because the dispersion of the nanowire diameter is mostly in the thermoelectric range [30,37]. Two very important issues have been faced for the fabrication of thermoelectric devices based on vertical silicon nanowire forests.

The first issue is related with the doping of the nanowires, because the MACE process is reliable only on slightly doped silicon substrates [80,81], with doping concentrations very low with respect to those for optimal thermoelectric properties. However, a thermal diffusion doping step can be performed after the nanowire fabrication [82,83,84]. The second issue is the fabrication of contacts for the electrical and thermal transport. In particular, the bottom of the nanowires is contacted because they are anchored to the silicon substrate, the top can be contacted by means of copper electrodeposition [85]. Very high power output density, of the order of μW/(cm${}^{2}$ K${}^{2}$) [34] have been achieved with p${}^{+}$ nanowire forest. This power has been measured on “macroscopic” samples of several mm${}^{2}$ of surface.

## 4. Conclusions

It is well assessed, on the basis of a large number of experimental results, that the thermal conductivity of silicon nanowires/nanostructures is strongly reduced with respect to the bulk status. This fact, together with the high power factor of silicon, which increases on a large temperature range, makes silicon one of the best materials for thermoelectric applications. Combining experimental measurements published by the more recent works, at first we show that the figure of merit of monocrystalline silicon nanostructures is very high in a large temperature range, and overcomes 1 for temperatures greater than 700 K. The main drawback is that the use of silicon for thermoelectric applications must get through nanofabrication issues. An advancement of these technologies can easily bring to on-chip all-silicon thermoelectric devices, to be used for microharvesting/scavenging. As a second point of our paper, we show the feasibility of macroscopic devices based on large collections of monocrystalline silicon nanostructures, reporting the fabrication processes based on top-down approaches and showing SEM micrographs of demonstrators.

These techniques open the way for a rapid application and commercialization at an industrial level of all-silicon devices for the direct conversion of heat into electrical power.
